# PIONEER-Panc: a platform trial for phase II randomized investigations of new and emerging therapies for localized pancreatic cancer

**DOI:** 10.1186/s12885-021-09095-7

**Published:** 2022-01-03

**Authors:** Julia E. Douglas, Suyu Liu, Junsheng Ma, Robert A. Wolff, Shubham Pant, Anirban Maitra, Eric P. Tamm, Priya Bhosale, Matthew H. G. Katz, Gauri R. Varadhachary, Eugene J. Koay

**Affiliations:** 1 Department of Radiation Oncology, The University of Texas MD Anderson Cancer Center, 1220 Holcombe Boulevard, MS97, Houston, TX 77030 USA; 2 Department of Biostatistics, The University of Texas MD Anderson Cancer Center, Houston, TX USA; 3 Department of Gastrointestinal Medical Oncology, The University of Texas MD Anderson Cancer Center, Houston, TX USA; 4 Sheikh Ahmed Center for Pancreatic Cancer Research, The University of Texas MD Anderson Cancer Center, Houston, TX USA; 5 Department of Pathology, The University of Texas MD Anderson Cancer Center, Houston, TX USA; 6 Department of Diagnostic Radiology, The University of Texas MD Anderson Cancer Center, Houston, TX USA; 7 Department of Surgical Oncology, The University of Texas MD Anderson Cancer Center, Houston, TX USA

**Keywords:** Pancreatic cancer, PIONEER, Phase II, Randomized platform trial, Resectable, Borderline resectable, Locally advanced, FOLFIRINOX, Gemcitabine/nab-paclitaxel, Pancreatic ductal adenocarcinoma (PDAC)

## Abstract

**Background:**

Personalized and effective treatments for pancreatic ductal adenocarcinoma (PDAC) continue to remain elusive. Novel clinical trial designs that enable continual and rapid evaluation of novel therapeutics are needed. Here, we describe a platform clinical trial to address this unmet need.

**Methods:**

This is a phase II study using a Bayesian platform design to evaluate multiple experimental arms against a control arm in patients with PDAC. We first separate patients into three clinical stage groups of localized PDAC (resectable, borderline resectable, and locally advanced disease), and further divide each stage group based on treatment history (treatment naïve or previously treated). The clinical stage and treatment history therefore define 6 different cohorts, and each cohort has one control arm but may have one or more experimental arms running simultaneously. Within each cohort, adaptive randomization rules are applied and patients will be randomized to either an experimental arm or the control arm accordingly. The experimental arm(s) of each cohort are only compared to the applicable cohort specific control arm. Experimental arms may be added independently to one or more cohorts during the study. Multiple correlative studies for tissue, blood, and imaging are also incorporated.

**Discussion:**

To date, PDAC has been treated as a single disease, despite knowledge that there is substantial heterogeneity in disease presentation and biology. It is recognized that the current approach of single arm phase II trials and traditional phase III randomized studies are not well-suited for more personalized treatment strategies in PDAC. The PIONEER Panc platform clinical trial is designed to overcome these challenges and help advance our treatment strategies for this deadly disease.

**Trial registration:**

This study is approved by the Institutional Review Board (IRB) of MD Anderson Cancer Center, IRB-approved protocol 2020-0075. The PIONEER trial is registered at the US National Institutes of Health (ClinicalTrials.gov) NCT04481204.

**Supplementary Information:**

The online version contains supplementary material available at 10.1186/s12885-021-09095-7.

## Background

Approximately 20-30% of patients with pancreatic ductal adenocarcinoma (PDAC) present with resectable or borderline resectable PDAC, and an additional 20-30% present with locally advanced disease. The focus of most drug development efforts is directed towards patients with metastatic disease. There are few trials that address novel therapeutics non-metastatic PDAC stages, and fewer still that conduct extensive correlative studies to elucidate biological underpinnings of response or resistance to specific therapies. The emergence and acceptance of neoadjuvant, pre-operative approaches to localized PDAC has created an opportunity to evaluate novel therapies in earlier stage disease, potentially helping advance therapeutic strategies for all stages of PDAC.

One reason for the growing use of neoadjuvant treatment of localized PDAC is that the disease is generally thought to be a systemically spread in most patients at the time of diagnosis, even though metastases are not evident on diagnostic imaging. Indeed, many patients who undergo upfront surgery develop distant metastasis at a high rate within 6 months. This fact underscores the need for better selection for surgical management and provides a sound rationale for pre-operative therapy. The neoadjuvant approach provides the advantages of treatment of micrometastatic disease, decreased R1 resection, and selection of patients who are fit for operation [1, 2]. Recently, the PREOPANC study suggested an improvement in disease free survival for patients who underwent neoadjuvant therapy as compared to upfront surgery for resectable/borderline resectable disease [3]. This study highlighted the potential beneficial role of neoadjuvant therapy for patients with radiologically localized disease at presentation. There remains a major need to improve systemic therapies for patients, however. Even in the experimental arm of PREOPANC that received neoadjuvant therapy, the median survival was only 16.0 months, as compared to 14.3 months in the immediate surgery arm. Here, we detail a multi-institutional phase II randomized-controlled Bayesian platform trial for investigations of new and emerging therapies called PIONEER-Panc.

## Methods/design (Fig. 1)

### Trial design

#### Overview

This is a phase II study using a Bayesian platform design [4]. There are three clinical stage groups of localized pancreatic cancer: resectable, borderline resectable, and locally advanced disease. Further, for each stage group, we will divide the patients into treatment naïve and previously treated groups. The treatment naïve and previously treated groups will have experimental regimens designed specifically for them (Fig. 2), since those who have previous treatment will have been selected for a specific chemotherapy regimen prior to enrollment, which would differ from the treatment naïve group and possibly introduce bias if the two groups were combined. Practically speaking, allowance of patients with prior treatment will enable more robust accrual to our study, as some of the patients seen at the participating hospitals only come for radiation and/or surgery after receiving chemotherapy elsewhere. Another reason to keep the treatment naïve and previously treated groups separate is that we anticipate that there will be experimental agents that will only be combined with radiation. We do not plan to blind the patients or the physicians to receipt of the experimental agent.

**Fig. 1:**
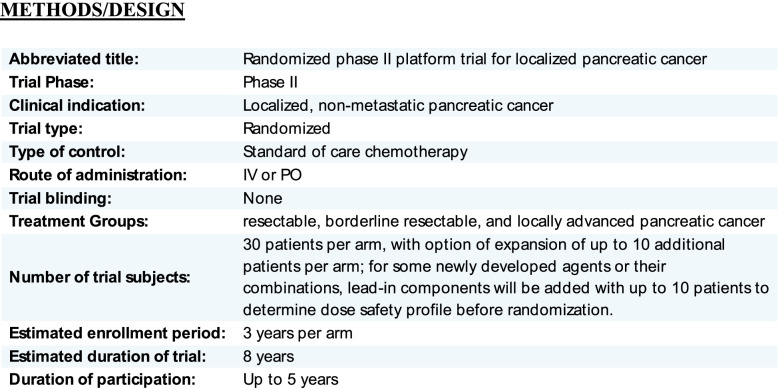
Trial Overview

**Fig. 2:**
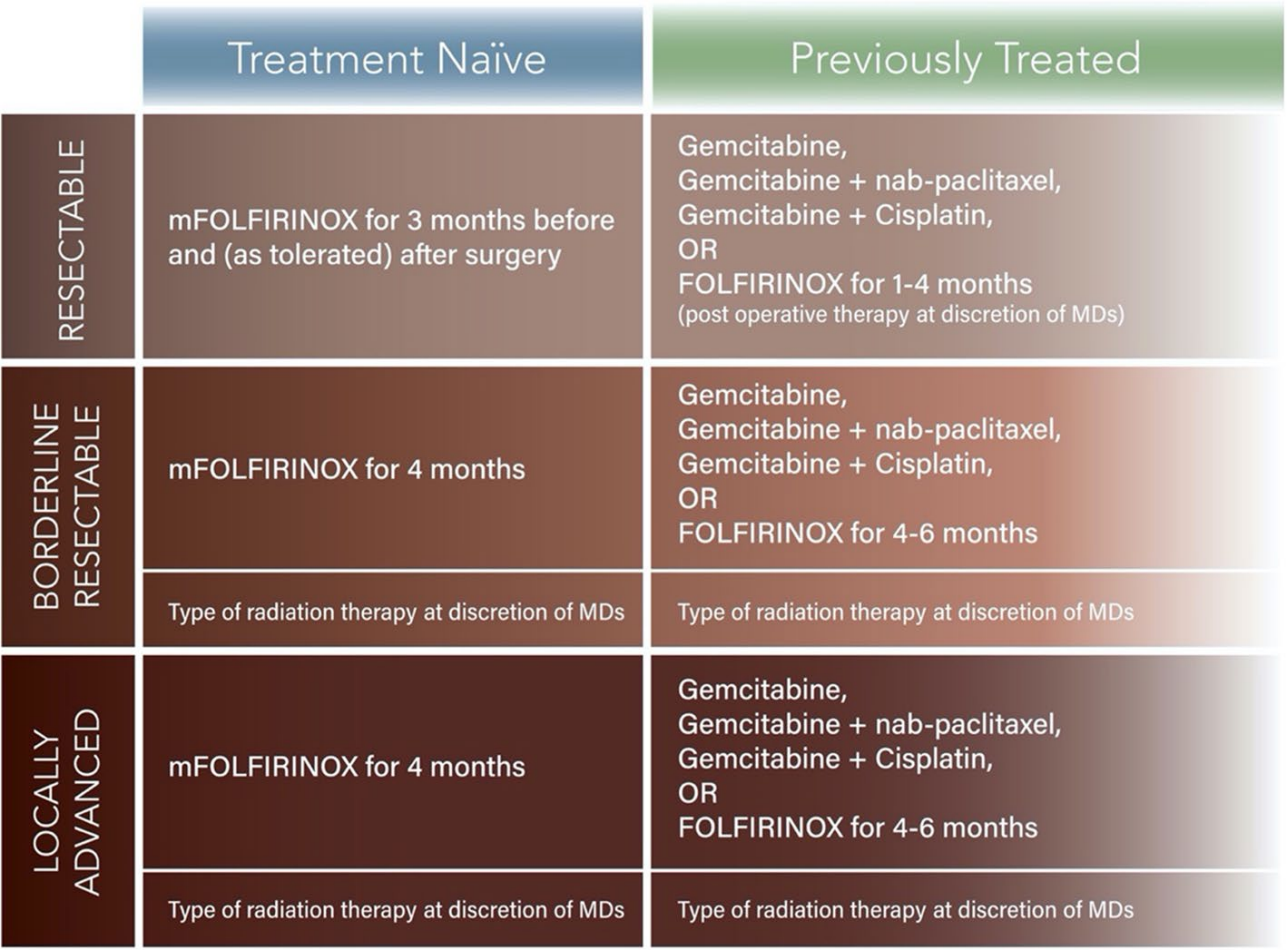
Summary of control arms for platform trial

Thus, we will have 3 staging groups, and each stage group will be divided into treatment naïve and previously treated cohorts. This gives a total of 6 different cohorts that will be studied (Fig. 2): (1) treatment naïve resectable PDAC, (2) previously treated resectable PDAC, (3) treatment naïve borderline resectable PDAC, (4) previously treated borderline resectable PDAC, (5) treatment naïve locally advanced PDAC, and (6) previously treated locally advanced PDAC. Each of the six subgroups will have a defined standard of care chemotherapy regimen for a control arm, serving as the basis of comparison. Each group may have one or more experimental arms. Experimental arms may be added to the platform during the study, and the effects of the experimental treatments will be compared with the controls for each group (Fig. 1).

For correlative studies, we will obtain pre- and post- treatment biopsies or tissues when feasible, perform serial blood draws for liquid biopsy analyses throughout treatment, collect radiomic data, and obtain quality of life measurements through validated questionnaires. Patients will be followed on a 1-3 month schedule during systemic therapy in general. During radiation therapy, they will be seen once a week, and once every 1-6 months in follow up after radiation. Patients with resectable and borderline resectable disease will be assessed for the primary endpoint of major pathological response rate [5], and the secondary endpoints of progression free survival and overall survival. Patients with locally advanced disease will be assessed for the primary endpoint of 6 month disease control rate (DCR), and the secondary endpoints of progression free survival and overall survival.

#### Trial diagram

**Fig. 3:**
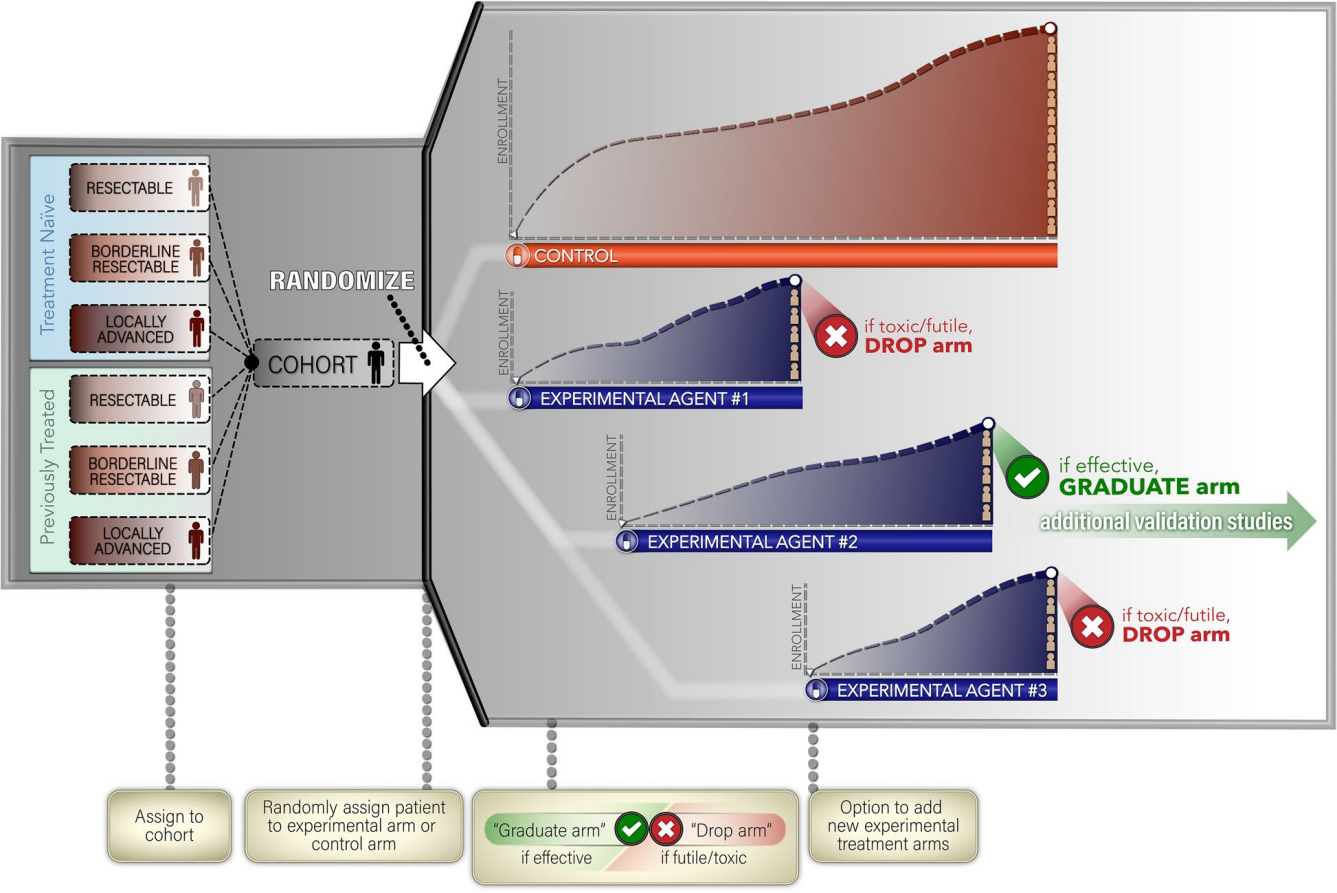
Trial schema and hypothetical randomization to the control and experimental arms

Eligible patients will be classified into one of the six cohorts defined by stage and treatment history as discussed previously; and upon enrollment into the trial, each patient will be randomized into control or experimental arms of the appropriate modules (Fig. 3). Accrual to the control arms for each cohort will continue over time until pre-specified limits are reached, while the randomization probability for the control arm will be gradually reduced when a new experiment arm is added. Although it is not expected that the control arm treatments will change over the time, we may change/update the control arm through amendments if forthcoming data collected from this trial or concurrent evidence from other trials/studies require us otherwise.

### Objectives and hypotheses

#### Impact of trial

The study will compare the effect of experimental treatment regimens, which may include compounds/biologicals, radiation techniques, and treatment devices, with the two most commonly employed preoperative regimens, systemic chemotherapy and radiation. We anticipate that these experimental treatments will provide signals for larger validation studies and the correlative science will give new insights into the biology of the disease, as well as strategies to personalize therapy.

#### Primary and secondary objectives

Primary and Secondary Objectives for resectable and borderline resectable groups (treatment naïve or previously treated):

1. Primary objective: To estimate major pathological response rate
2. Secondary objectives: To measure progression free survival and overall survival

Primary and Secondary Objectives for locally advanced groups (treatment naïve or previously treated):

1. Primary objective: To estimate 6-month disease control rate
2. Secondary objectives: To measure progression free survival and overall survival

#### Exploratory objectives in patients also consented for correlative studies

1. To demonstrate response through exosome and circulating tumor DNA
2. To associate prognosis of patients with baseline and follow-up quantitative CT image-based analysis
3. To associate clinical and pathological outcomes of patients with changes in radiomic measurements

### Patient selection

#### Disease status criteria table

**Fig. 4:**
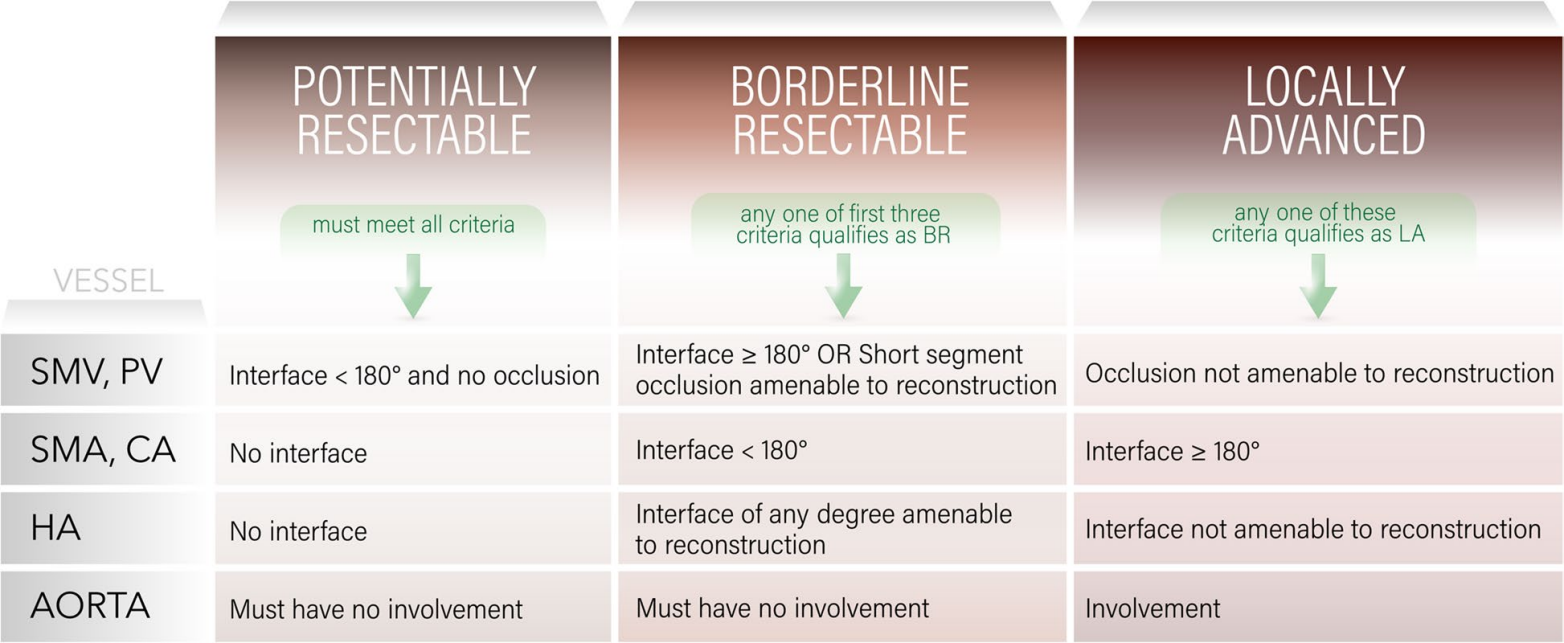
Disease Status Criteria

To be considered part of each subgroup, the patient must have as noted: all characteristics of the potentially resectable subgroup, one or more of the characteristics of the borderline resectable subgroup without any from the locally advanced subgroup, or any of the characteristics from the locally advanced subgroup (Fig. 4).

#### Eligibility and ineligibility criteria

**Fig. 5:**
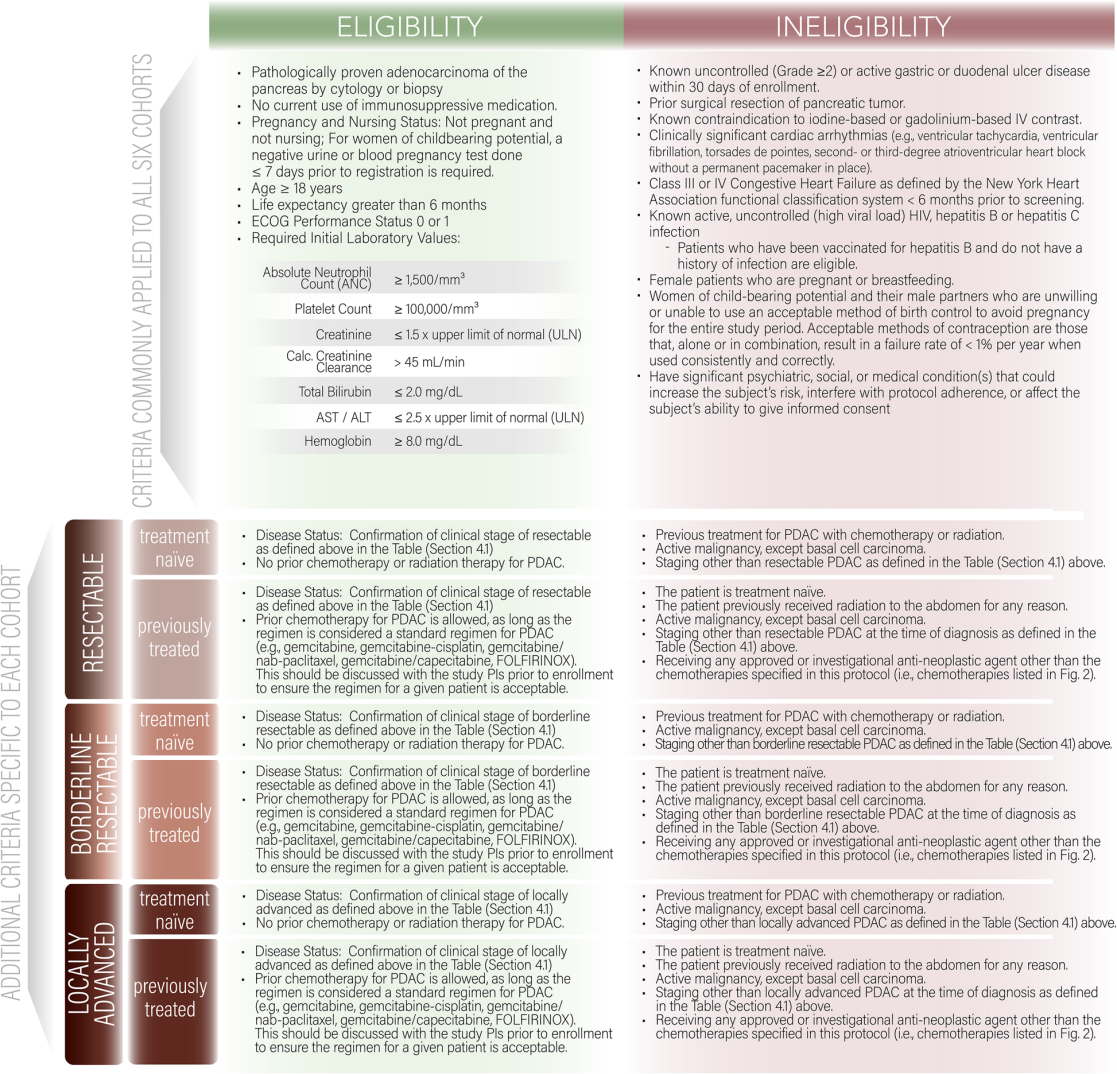
Eligibility and Ineligibility Criteria

The general criteria for the platform trial are shown in Fig. 5 and for each of the six cohorts, respectively.

### Treatment calendars

**Fig. 6:**
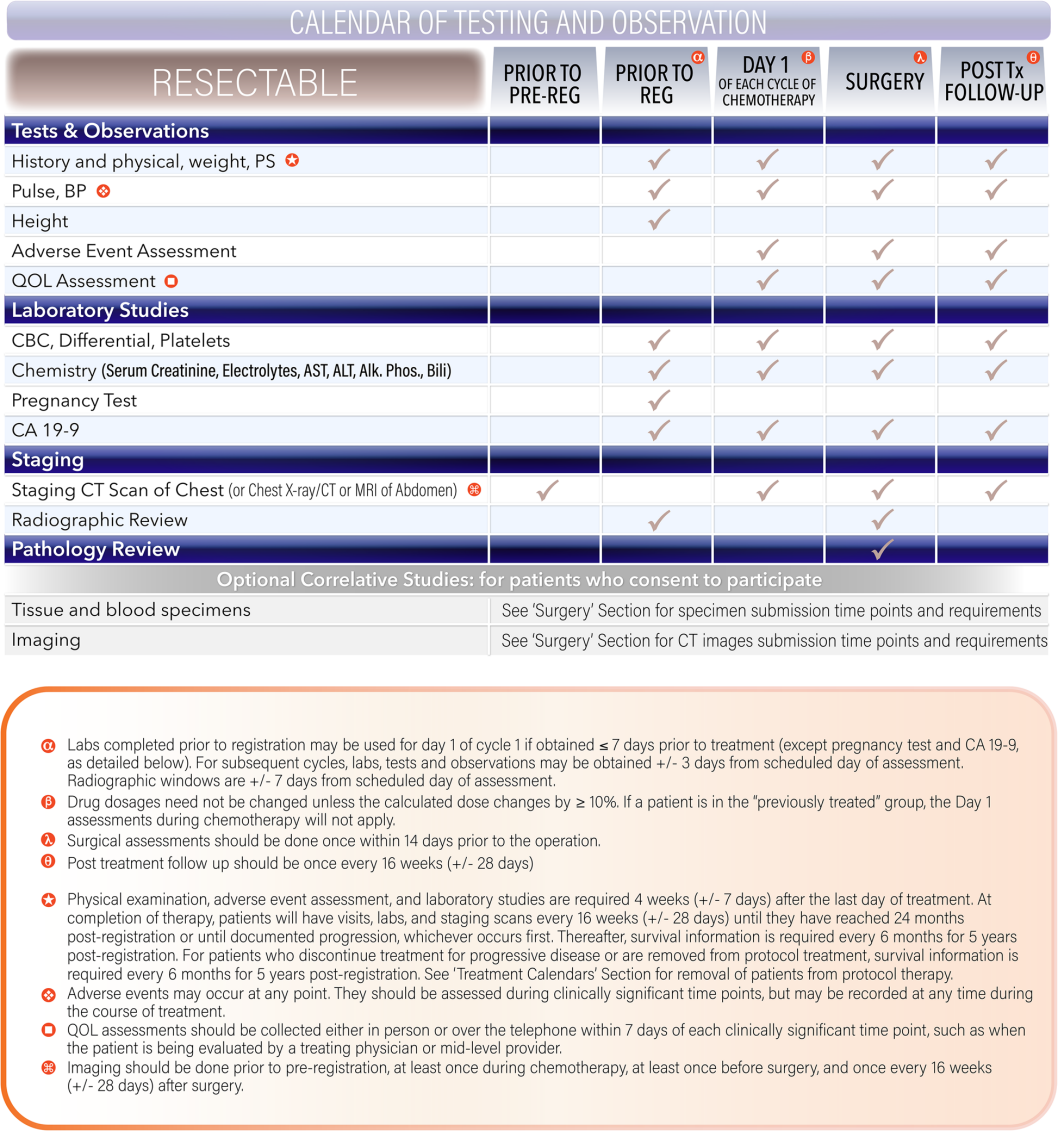
Calendar of Testing and Observation (Resectable)

The following study calendar (Fig. 6) represents general guidelines for assessments related to the protocol. Figure 6 relates to the resectable group in particular; the calendars for other groups may differ slightly. Specific modules may have additional requirements.

Physical examination, adverse event assessment, and laboratory studies are required 4 weeks (+/− 7 days) after the last day of treatment. At completion of therapy, patients will have visits, labs, and staging scans every 16 weeks (+/− 28 days) until they have reached 24 months post-registration or until documented progression, whichever occurs first. Thereafter, survival information is required every 6 months for 5 years post-registration. For patients who discontinue treatment for progressive disease or are removed from protocol treatment, survival information is required every 6 months for 5 years post-registration. Similar calendars are in the protocol document for patients with borderline resectable or locally advanced disease.

### Diagnostic imaging

The CT scans will be standard of care pancreatic protocol scans with contrast [6]. A pancreatic protocol CT involves iodine-based IV contrast agent protocols to reach optimal differentiation of normal pancreatic tissue from lesions, along with sufficient visualization of the pancreatic vessels to allow for local staging. The study will use a dual-phase CT acquisition after IV contrast medium administration at a flow rate of 3 – 5 mL/s for optimal pancreatic CT enhancement [7].

### Chemotherapy

The standard of care regimens for PDAC include gemcitabine/nab-paclitaxel and mFOLFIRINOX. In this adaptive trial, we will use mFOLFIRINOX as the standard of the control arms for “treatment naïve” patients with “potentially resectable”, “borderline resectable”, and “locally advanced” disease. For “previously treated” groups, we will include patients in the trial if they received gemcitabine, gemcitabine/cisplatin, gemcitabine/nab-paclitaxel, gemcitabine/capecitabine, or FOLFIRINOX according to accepted guidelines below. Patients in the “previously treated” groups will be enrolled after the appropriate amount of standard chemotherapy is completed and restaging is performed. They will then be randomized to experimental or control arms as appropriate to their clinical staging at the diagnosis.

*Dosing schedules and modifications: All commercially supplied drugs will follow the manufacturer-provided labeling with respect to its storage and stability, preparation, handling, and administration. Decisions regarding dose modifications, and delays will be made for each patient at the discretion of the attending physician. All other treatments, including chemotherapy pre-medications, will be determined at the discretion of the attending physician.*

### Radiotherapy

Patients may receive radiation therapy as part of this trial. Techniques of radiation may include stereotactic body radiation therapy, hypofractionated radiation therapy [8], and standard fractionation radiation therapy [9] with concurrent chemotherapy. The delivery technique may be tailored to a given experimental arm. In the control arm, the use of radiation will be standard in borderline resectable disease. In patients with resectable disease, it is not required but may be used in the control arm if a physician deems it appropriate.

### Surgery

Surgical resection of the primary tumor and regional lymph nodes in the absence of disease progression 4-8 weeks following chemotherapy and/or radiation. Surgical quality assurance will be performed in the same manner as Alliance A021501 [10].

### Correlative studies

Biospecimens and imaging will be collected as part of an IRB approved protocol for biospecimen collection (PA11-0670). The informed consent document (see supplementary materials) for this adaptive trial will clearly state that the patient is co-enrolling on PA11-0670 if they agree to any correlative study for this adaptive clinical trial. Here, we describe how the biospecimens and imaging will be analyzed.

#### Liquid biopsies

To demonstrate response through exosome and circulating free tumor cells. We will obtain serial liquid biopsies at clinically significant time points and derive exosome and circulating free tumor cells as per established protocols [11]. The levels of exosomes and circulating free tumor cells will be associated with clinical outcomes and pathological responses that are observed during the course of the trial.

#### Imaging

1. To associate prognosis of patients with baseline quantitative CT image-based analysis. CT images will be obtained for patients as part of standard of care at clinically significant time points. We have previously demonstrated that measurements of baseline enhancement (e.g., area under the enhancement curve) and the morphology of the PDAC tumors (e.g., presence or absence of a distinct border, called high or low delta PDAC, respectively) are associated with stromal, genetic, physical, and clinical characteristics of the disease [12, 13]. We will validate these associations in this study.
2. To associate clinical and pathological outcomes of patients with changes in radiomic measurements. We previously showed that the development of a sharpened interface at the tumor/parenchymal border was associated with longer PFS and OS, compared to blurring of the tumor/parenchymal interface [14]. We will associate this radiographic feature after neoadjuvant therapy with pathological measurements and clinical outcomes on the trial.

### Adverse events

The prompt reporting of adverse events is the responsibility of each investigator engaged in clinical research, as required by Federal Regulations. Adverse events must be described and graded using the terminology and grading categories defined in the NCI’s Common Terminology Criteria for Adverse Events (CTCAE), Version 5.0. The CTCAE is available at ctep.cancer.gov/protocolDevelopment/electronic_applications/ctc.htm. Attribution to protocol treatment for each adverse event must be determined by the investigator and reported on the required forms. Please refer the NCI Guidelines: Adverse Event Reporting Requirements for further details on AE reporting procedures. All toxicities will be managed according to institutional algorithms as available, or per best practice.

### Statistical considerations

In this study, we have six cohorts of patients based on clinical staging and treatment history as described in Trial design section. For each cohort, a Bayesian phase II platform design will be used to evaluate a sequence of experimental agents. The goal of the study is to collect data and to estimate the efficacy of the experimental treatment, with relatively rapid readouts for go/no go decisions of therapeutics in early stage PDAC. The primary endpoint for patients with clinical staging of resectable or borderline resectable disease is the major pathological response (MPR) at 12 weeks since randomization, and for patients with clinical staging of locally advanced or unresectable disease is the 6-month DCR, defined as the proportion of patients without progression within 6 months of the treatment.

Based on the platform design, we are allowed to simultaneously randomize patients into multiple experimental treatment arms. For each disease cohort, we will start with one control and one experimental treatment arm, while additional experimental treatment arms can be open for enrollment during the study. We will start with equally randomizing patients into the control and experimental arms, and assume a new experimental agent arm may enter the study 6 months after the opening of an experimental arm. In order to maintain an active control during the study, we will keep randomizing patients into the control arm, while the randomization probability will be gradually reduced upon the number of patients treated in the control arm and the number of active experiment arms. Once the control arm has treated the prespecified number (e.g., *n* = 30) of patients, we will rescale the randomization probability so that more patients will be allocated into the experimental arms. Specifically, the randomization probability for the control arm will be reduced to be 1/(1 + 3 m), and the randomization probability for each experimental arm will be 3/(1 + 3 m), where m is the number of active experimental arms in the study. That is, the randomization probability for control arm will be reduced to be 1/7 if there are 2 experimental arms in the study, and will be reduced to 1/10 if 3 experimental arms in the study, and etc. The randomization schema and the timeline of the experimental agent arms are illustrated in Fig. 3.

After an experimental arm has treated the planned 30 patients, we will compare it to the control arm to see if the experimental treatment has improved the MPR or DCR. The experimental treatment will be claimed to be successful if:

1 $$\Pr \Big({\pi}_e>{\pi}_c+0.05\ \left|\mathrm{data}\right)>{\theta}_t,$$

where *π*_*e*_ and *π*_*c*_ are the MPR or DCR for the experimental arm and control arm, respectively. We choose to use Bayesian paradigm, so that we can naturally incorporate our knowledge or assumption of MPR or DCR of the treatment arms using a Beta prior distribution. In this study non-informative prior distributions are assumed for *π*_*e*_ and *π*_*c*_, i.e., *π*_*e*_ ~ Beta(1,1), and *π*_*c*_ ~Beta(1,1), respectively. The Beta (1,1) distribution can be thought as a study of 2 patients, and only 1 response observed. It has a mean value of 0.50 with a wide 95% confidence interval of (0.02, 0.98). Since we do not have any definite knowledge of the efficacy of the treatment arms, this non-informative prior only expresses vague information about the primary endpoint. When the maximum sample size is reached for an experimental arm, we will calculate the posterior probability (PP) in (1) and compare it with the threshold *θ*_*t*_. If the PP is greater than *θ*_*t*_, the experiment arm is deemed to be successful compared to the control arm. The threshold *θ*_*t*_ needs to be calibrated so that the trial will have the false postitive rate (type I error) no more than 15%. For those experimental treatments that “graduate” from the study and appear to outperform the control arm, a steering committee of the co-principal investigators and scientific advisors will meet to decide whether to expand the experimental arm (e.g., up to *n* = 10 additional patients) to confirm the findings.

The randomization will be conducted using the Clinical Trial Conduct (CTC) website (https://biostatistics.mdanderson.org/ClinicalTrialConduct), which is housed on a secure server at MDACC and maintained by the MDACC Department of Biostatistics. Access to the website will be gained through usernames and passwords provided by the MDACC Department of Biostatistics to the clinical team. Training on the use of the CTC website to randomize patients on the study will be provided by the biostatistical collaborators. The study will be monitored by the MD Anderson Data Safety Monitoring Board (DSMB).

#### Simulation studies

The planned sample size is 30 for each experimental arm. We assume a new experimental arm may enter the study 6 months after the opening of the previous experimental arm. The values of threshold *θ*_*t*_ are 0.67 and 0.72 for MPR and DCR, repectively.

For each scenario, 1000 trials were simulated and probability of claiming success is summarized as the proportion of trials having the posterior probability greater than the shreshold value *θ*_*t*_ . Specifically, for each simulated trial we calculate the posterior probability Pr(*π*_*e*_ > *π*_*c*_ + 0.05 |data) and compare it with the threshold value of *θ*_*t*_ (e.g., 0.67) using the decision rule (1). The probability of claiming success is the proportion of trials that meet (1). Note that, the experimental treatment agents are chosen to be included for their evidence of efficacy shown in previous studies, and therefore no early stopping rule for efficacy will be implemented.

#### Major pathological response rate

We assumed the MPR being 13% for the control arm based on historical data from MD Anderson [15]. We anticipate some of the experimental arms will have improved response rates of 19.5% (1.5 fold), 26% (2 fold) and 30%, respectively. The simulation results are summarized in Table 1 below. With *θ*_*t*_ = 0.67, the type I error is less or equal to 15%. That is, if an experimental arm (e.g., Experiment Arm 3) has the same response rate as the control arm, the probability of claiming its success is less than or equal to 15%. In this setting, we will have 67.6% power to claim the success of an experimental arm with response rate being 26% (Experiment Arm 2). If the response rate is 30% for an experimental arm (Experiment Arm 4), the power will be 79.4%.

**Table 1:** Operating characteristics for the platform design with primary endpoint of major pathological response (MPR)

	Control Arm	*n = 30 for experimental arms*			
		Experiment Arm 1	Experiment Arm 2	Experiment Arm 3	Experiment Arm 4
**True MPR**	0.130	0.195	0.260	0.130	0.300
**Number of patients**	42.6	30.0	30.0	30.0	30.0
**Start Time (months)**	0	0	6	12	18
**Probability of claiming success**	–	0.384	0.676	0.148	0.800

#### Six-month disease control rate

We assumed the 6-month DCR being 70% for the control arm based on historical data for locally advanced disease at MD Anderson [16]. We anticipate some of the experimental arms will have an improved DCR of 90%. The simulation results are summarized in Table 2 below. With *θ*_*t*_ = 0.72, the type I error is less or equal to 15%. That is, if an experimental arm (e.g., Experiment Arm 1) has the same DCR as the control arm, the probability of claiming its success is less than or equal to 15%. In this setting, we will have 85.6% power to claim the success of an experimental arm with DCR being 90% (Experiment Arm 3).

**Table 2:** Operating characteristics for the platform design with primary endpoint of disease control rate (DCR)

	Control Arm	*n = 30 for experimental arms*			
		Experiment Arm 1	Experiment Arm 2	Experiment Arm 3	Experiment Arm 4
**True DCR**	0.70	0.70	0.80	0.90	0.95
**Number of patients**	42.6	30.0	30.0	30.0	30.0
**Start Time (months)**	0	0	6	12	18
**Probability of claiming success**	–	0.148	0.441	0.833	0.964

#### Safety lead-in phase and toxicity monitoring

For some newly developed agents or their combinations, a safety lead-in phase will be applied before the randomization. We will apply BOIN design to determine the dose/schedule for the randomization part [4, 17]. The details will be described in the specific protocol for that experimental arm.

Additionally, a Bayesian toxicity monitoring rule may be also implemented for experimental arms that are deemed necessary by the investigators, the scientific advisors and the statisticians. The events will include grade 3 or higher hematological, gastrointestinal or any other toxicities that are at least possibly related to treatment during the time window from the treatment initiation till 30 days after the treatment ends, according to Common Terminology Criteria for Adverse Events (CTCAE) v4.0. Let *π*_*tox*_ be the toxicity probability with a prior distribution of Beta(0.6,1.4), then if Pr (*π*_*tox*_ > 0.3]) > 0.8, we will terminate the experimental arm early. Patients will be monitored in cohorts of size 10. Based on these assumptions and monitoring conditions, we will early stop the experiment arm if we observe [# patients experiencing toxicity] / [#patients being treated] > = 5/10, 8/20, or 12/30. The operating characteristics are shown in Table 3. The in-house software (https://trialdesign.org/one-page-shell.html#BTOX) was used to generate the toxicity boundaries and operating characteristics.

**Table 3:** Operating characteristics for toxicity monitoring

True toxicity probability	Early stopping probability	Average sample size
0.2	0.052	29.2
0.3	0.273	25.8
0.4	0.623	20.1
0.5	0.884	14.9
0.6	0.982	11.8

#### Analysis plan

Patients’ demographic and clinical characteristics will be summarized using descriptive statistics of count frenquecies, percentages, means, standard deviations, medians and ranges. Associations between groups will be assessed using the Chi Square test or Fisher’s exact test for categorical variables, and t-test or Wilcoxon rank sum test for continuous variables. For primary outcome variables, we will estimate MPR and DCR along with their 95% exact confidence intervals (CIs) using the Clopper and Pearson method. Among 30 patients treated, assuming 6 patients experience MPR, the 95% CIs will be (0.077, 0.386); and assuming 27 out of 30 patients have disease controlled, the 95% confidence interval of DCR will be (0.735, 0.979). Generalized linear regression models will be explored to evaluate the associations between the endpoint of MPR/DCR and covariates of interest.

Overall survival time and progression free survival time will be calculated for each patient. The overall survival time is defined as the time duration from treatment start till death or last follow-up if the patient is alive. Progression free survival time is defined as the time period from the date of treatment initiation to the date of disease progression, recurrence after surgery or death from any cause whichever occurs first. The distributions of overall survival and progression free survival will be estimated using the Kaplan-Meier method. Comparisons of these time-to-event endpoints by important covariate subgroups will be made using the log-rank tests. Cox proportional hazards regression models will be explored to evaluate the associations between the time-to-event endpoints and covariates of interest.

In addition, for exploratory analysis, we may apply the Bayesian classification and information sharing method in the data analysis, especially when we assess the potential predictive marker effects on treatment. The hierarchical model will allow us to borrow strength across similarly performed arms. It will provide a more efficient and more powerful way to estimate and test the outcomes, especially when we have several experimental arms showing similar clinical benefit compared the control arm.

## Discussion

This Bayesian platform adpative clinical trial design has several advantages over a traditional clinical trial design. The main advantages include (1) efficiencies in resource allocation over time, (2) the ability to adaptively randomize patients according to pre-specified criteria (clinical endpoints, number of open arms), and (3) uniform collection of correlative studies spanning multiple therapeutic arms. We adaptively eliminate the ineffective or overly toxic agent during the trial, and graduate the efficacious agents to the next phase of development. With regards to the efficiencies gained, adaptive trial designs enable the comparison of multiple experimental treatment arms to a single control arm, eliminating the need to repeat the control arm that would otherwise be needed if each experimental arm were conducted in a traditional randomized Phase II design [18]. Since this is a Phase 2 design, the experimental agents that we intend to include in the future are expected to have strong preclinical rationale, and ideally some preliminary signs of efficacy from prior Phase 1 studies.

While this study seeks innovative treatments for localized pancreatic cancer, another study exists that incorporates similar design characteristics but is focused on metastatic pancreatic cancer. The Precision Promise Platform Trial for Metastatic Pancreatic Cancer is an ongoing interventional clinical trial which utilizes a Phase 2/3 platform trial designed to evaluate multiple regimens for first and second metastatic patients [19].

Novel trial designs such as Precision Promise and PIONEER Panc aim to move the needle for patients with pancreatic cancer. Rapid, robust statistical designs are expected to help achieve the potential of personalized care for patients with this deadly disease.

## Supplementary Information

**Additional file 1.** Informed consent/authorization for participation in research with optional procedures.

## Data Availability

The datasets used and/or analysed during the current study are available from the corresponding author on reasonable request.

## Abbreviations

AE: Adverse event
BOIN: Bayesian Optimal Interval Design
BR: Borderline resectable
BRPC: Borderline Resectable Pancreatic Cancer
CA: Celiac Artery
CI: Confidence interval
CT: Computed tomography
CTC: Clinical Trial Conduct
CTCAE: Common Terminology Criteria for Adverse Events
DSMB: Data Safety Monitoring Board
DCR: Disease control rate
FOLFIRINOX: Fluorouracil Leucovorine, Irinotecan, Oxaliplatin
HA: Hepatic Artery
IV: Intravenous
LA: Locally advanced
LAPC: Locally Advanced Pancreatic Cancer
MDACC: MD Anderson Cancer Center
MPR: Major pathological response
NCI: National Cancer Institute
OS: Overall survival
PDAC: Pancreatic ductal adenocarcinoma
PFS: Progression-Free Survival
PV: Portal Vein
RS: Resectable
SMA: Superior Mesenteric Artery
SMV: Superior Mesenteric Vein
