# Supplementary material for: PIONEER-Panc: a platform trial for phase II randomized investigations of new and emerging therapies for localized pancreatic cancer
Source: BMC Cancer. 2022 Jan 3;22:14. doi: 10.1186/s12885-021-09095-7 (PMC8722115; doi:10.1186/s12885-021-09095-7)
Supplement: Supplementary file 1 — Additional file 1. Informed consent/authorization for participation in research with optional procedures. [file 12885_2021_9095_MOESM1_ESM.docx]

| 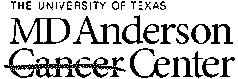 | **Informed Consent** |
| --- | --- |

INFORMED CONSENT**/**AUTHORIZATION FOR PARTICIPATION IN RESEARCH WITH OPTIONAL PROCEDURES

PIONEER-Panc: Phase II Investigations of New and Emerging Therapies for Pancreatic Cancer

2020-0075


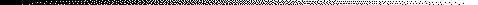


**Subtitle: Screening Consent**

**Study Chair:**  Eugene Koay

_________________________ _____________________________

Participant’s Name Medical Record Number

This is an informed consent and authorization form for a research study. It includes a summary about the study. A more detailed description of procedures and risks is provided after the summary.

**STUDY SUMMARY**

The goal of this clinical research study is to compare how effective different therapies are in controlling different types of pancreatic cancer. The safety of these therapies will also be studied.

The tests and procedures described in this screening consent will help the study doctor decide if you are eligible to take part in the study.

**This is an investigational study.**

Screening may show that you are eligible to take part in the study, in which the study treatment may help to control the disease. Future patients may benefit from what is learned. There may be no benefits for you in this study.

Your participation is completely voluntary. Before choosing to take part in this study, you should discuss with the study team any concerns you may have, including side effects, potential expenses, and time commitment.

You can read a full list of potential side effects below in the Possible Risks section of this consent.

You and/or your insurance provider will be responsible for any costs associated with the screening tests.

You may choose not to take part in this study. Instead of taking part in this study, you may choose to receive standard of care treatment. You may choose to receive other investigational therapy, if available. You may choose not to have treatment for cancer at all. In all cases, you will receive appropriate medical care, including treatment for pain and other symptoms of cancer.

**1. STUDY DETAILS**

**Screening Tests**

Signing this consent form does not mean that you will be able to take part in this study. The following screening tests will help the doctor decide if you are eligible:

- You will have a physical exam.
- You will have MRIs or CT scans of your chest, abdomen, and pelvis to check the status of the disease.
- Blood (about 4 tablespoons) will be drawn for routine tests.
- You will have an EKG to check your heart function.
- If you can become pregnant, urine will be collected for pregnancy testing. If the test is positive, blood (about ½ tablespoon) will be drawn to confirm the result. To take part in this study, you must not be pregnant.

The study doctor will discuss the screening test results with you. If the screening tests show that you are not eligible to take part in the study, you will not be enrolled. Other options will be discussed with you.

If you are found to be eligible to take part in this study, you will be assigned to a study group based on when you join this study. You will receive a separate consent form that describes the therapy being performed in the study group to which you are assigned.

Up to 105 participants will be enrolled in this study. All will take part at MD Anderson.

**2. POSSIBLE RISKS**

While on this study, you are at risk for side effects. These side effects will vary from person to person and depends on the local therapy choice and location of the tumor(s). The more commonly occurring side effects are listed in this form, as are rare but serious side effects. You should discuss these with the study doctor. You may also want to ask about uncommon side effects that have been observed in small numbers of patients but are not listed in this form. Many side effects go away shortly after the procedure, but in some cases side effects may be serious, long-lasting or permanent, and may even result in hospitalization and/or death.

Tell the study staff about any side effects you may have, even if you do not think they are related to the procedure.

**Blood draws** may cause pain, bleeding, and/or bruising. You may faint and/or develop an infection with redness and irritation of the vein at the site where blood is drawn. Frequent blood collection may cause anemia (low red blood cell count), which may create a need for blood transfusions.

This study may involve unpredictable risks to the participants.

**3. COSTS AND COMPENSATION**

If you suffer injury as a direct result of taking part in this study, MD Anderson health providers will provide medical care. However, this medical care will be billed to your insurance provider or you in the ordinary manner. You will not be reimbursed for expenses or compensated financially by MD Anderson for this injury. You may also contact the Chair of MD Anderson’s IRB at 713-792-6477 with questions about study-related injuries. By signing this consent form, you are not giving up any of your legal rights.

Certain tests, procedures, and/or drugs that you may receive as part of this study may be without cost to you because they are for research purposes only. However, your insurance provider and/or you may be financially responsible for the cost of care and treatment of any complications resulting from the research tests, procedures, and/or drugs. Standard medical care that you receive under this research study will be billed to your insurance provider and/or you in the ordinary manner. Before taking part in this study, you may ask about which parts of the research-related care may be provided without charge, which costs your insurance provider may pay for, and which costs may be your responsibility. You may ask that a financial counselor be made available to you to talk about the costs of this study.

Samples that are collected from you in this study may be used for the development of treatments, devices, new drugs, or patentable procedures that may result in commercial profit.

There are no plans to compensate you for any patents or discoveries that may result from your participation in this research.

You will receive no compensation for taking part in this study.

**Additional Information**

4. You may ask the study chair (Dr. Eugene Koay, at 713-563-2381) any questions you have about this study. You may also contact the Chair of MD Anderson's Institutional Review Board (IRB - a committee that reviews research studies) at 713-792-6477 with any questions that have to do with this study or your rights as a study participant.

5. You may choose not to take part in this study without any penalty or loss of benefits to which you are otherwise entitled. You may also withdraw from participation in this study at any time without any penalty or loss of benefits. If you decide you want to stop taking part in the study, it is recommended for your safety that you first talk to your doctor. If you withdraw from this study, you can still choose to be treated at MD Anderson.

6. This study or your participation in it may be changed or stopped without your consent at any time by the study chair, the U.S. Food and Drug Administration (FDA), the Office for Human Research Protections (OHRP), or the IRB of MD Anderson.

7. You will be informed of any new findings or information that might affect your willingness to continue taking part in the study, including the results of all of your standard tests performed as part of this research, and you may be asked to sign another informed consent and authorization form stating your continued willingness to participate in this study.

You may receive the results of the blood biomarker, imaging, and genetic tests done during this study.

8. MD Anderson may benefit from your participation and/or what is learned in this study.

**Future Research**

**Data**

Your personal information is being collected as part of this study. These data may be used by researchers at MD Anderson and the company providing the drug, and/or shared with other researchers and/or institutions for use in future research.

**Samples**

Samples (such as blood and/or tissue) are being collected from you as part of this study. Researchers at MD Anderson may use any leftover samples that are stored at MD Anderson in future research.

Before being used or shared for future research, every effort will be made to remove your identifying information from any data and/or research samples. If all identifying information is removed, you will not be asked for additional permission before future research is performed.

In some cases, all of your identifying information may not be removed before your data or research samples are used for future research. If future research is performed at MD Anderson, the researchers must get approval from the

Institutional Review Board (IRB) of MD Anderson before your data and/or research samples can be used. At that time, the IRB will decide whether or not further permission from you is required. The IRB is a committee of doctors, researchers, and community members that is responsible for protecting study participants and making sure all research is safe and ethical.

If this research is not performed at MD Anderson, MD Anderson will not have oversight of any data and/or samples

**Genetic Research**

Samples collected from you as part of this study may be used for genetic research, which may include whole genome sequencing. Whole genome sequencing is a type of testing in which researchers study your entire genetic makeup (DNA). This may help researchers learn how changes in the ordering of genes may affect a disease or response to treatment.

**Authorization for Use and Disclosure of Protected Health Information (PHI):**

A. During the course of this study, MD Anderson will be collecting and using your PHI, including identifying information, information from your medical record, and study results. For legal, ethical, research, and safety-related reasons, your doctor and the research team may share your PHI with:

- Federal agencies that require reporting of clinical study data (such as the FDA, National Cancer Institute [NCI], and OHRP)
- The IRB and officials of MD Anderson
- Study monitors and auditors who verify the accuracy of the information
- Individuals who put all the study information together in report form

The results of this research may be published. However, your name and other identifying information will be kept confidential. Your information will be protected from disclosure to others to the extent required by law. Complete privacy cannot be promised.

B. Signing this consent and authorization form is optional but you cannot take part in this study or receive study-related treatment if you do not agree and sign.

C. MD Anderson will keep your PHI confidential when possible (according to state and federal law). However, in some situations, the FDA could be required to reveal the names of participants.

Once disclosed outside of MD Anderson, federal privacy laws may no longer protect your PHI.

D. The permission to use your PHI will continue indefinitely unless you withdraw your authorization in writing. Instructions on how to do this can be found in the MD Anderson Notice of Privacy Practices (NPP) or you may contact the Chief Privacy Officer at 713-745-6636. If you withdraw your authorization, you will be removed from the study and the data collected about you up to that point can be used and included in data analysis. However, no further information about you will be collected.

E. A description of this clinical trial will be available on http://www.ClinicalTrials.gov, as required by U.S. Law. This Web site will not include information that can identify you. At most, the Web site will include a summary of the results. You can search this Web site at any time.

# CONSENT/AUTHORIZATION

I understand the information in this consent form. I have had a chance to read the consent form for this study, or have had it read to me. I have had a chance to think about it, ask questions, and talk about it with others as needed. I give the study chair permission to enroll me on this study. By signing this consent form, I am not giving up any of my legal rights. I will be given a signed copy of this consent document.

**________________________________________ _______**

SIGNATURE OF PARTICIPANT DATE

# _________________________________

PRINTED NAME OF PARTICIPANT

# LEGALLY AUTHORIZED REPRESENTATIVE (LAR)

The following signature line should only be filled out when the participant does not have the capacity to legally consent to take part in the study and/or sign this document on his or her own behalf.

**_____________________________________________________ _________**

SIGNATURE OF LAR DATE

#

PRINTED NAME and RELATIONSHIP TO PARTICIPANT

# WITNESS TO CONSENT

I was present during the explanation of the research to be performed under Protocol 2020-0075.

**_____________________________________________________ _________**SIGNATURE OF WITNESS TO THE VERBAL CONSENT DATE
PRESENTATION (OTHER THAN PHYSICIAN OR STUDY CHAIR)

A witness signature is only required for vulnerable adult participants. If witnessing the assent of a pediatric participant, leave this line blank and sign on the witness to assent page instead.

PRINTED NAME OF WITNESS TO THE VERBAL CONSENT

# PERSON OBTAINING CONSENT

I have discussed this research study with the participant and/or his or her authorized representative, using language that is understandable and appropriate. I believe that I have fully informed this participant of the nature of this study and its possible benefits and risks and that the participant understood this explanation.

# ______________________________________________ _________

PERSON OBTAINING CONSENT DATE

**______________________________________________**

PRINTED NAME OF PERSON OBTAINING CONSENT

# TRANSLATOR

I have translated the above informed consent as written (without additions or subtractions) into and assisted the people

(Name of Language)

obtaining and providing consent by translating all questions and responses during the consent process for this participant.

**_____________________ _______________________________ ___________**

NAME OF TRANSLATOR SIGNATURE OF TRANSLATOR DATE


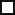
 Please check here if the translator was a member of the research team. (If checked, a witness, other than the translator, must sign the witness line below.)

**____________________________________________________ ___________**

SIGNATURE OF WITNESS TO THE VERBAL TRANSLATION DATE
(OTHER THAN TRANSLATOR, PARENT/GUARDIAN,
OR STUDY CHAIR)
